# Propionyl-L-carnitine Corrects Metabolic and Cardiovascular Alterations in Diet-Induced Obese Mice and Improves Liver Respiratory Chain Activity

**DOI:** 10.1371/journal.pone.0034268

**Published:** 2012-03-23

**Authors:** Carmen Mingorance, Lucie Duluc, Matthieu Chalopin, Gilles Simard, Pierre-Henri Ducluzeau, Maria Dolores Herrera, Maria Alvarez de Sotomayor, Ramaroson Andriantsitohaina

**Affiliations:** 1 Department of Pharmacology, School of Pharmacy, University of Sevilla, Sevilla, Spain; 2 LUNAM Université, Anger, France; 3 INSERM U1063, Angers, France; 4 Université d'Angers, CHU Angers, Department of Biochemistry, Angers, France; Sapienza University of Rome, Italy

## Abstract

**Aims:**

Obesity is a primary contributor to acquired insulin resistance leading to the development of type 2 diabetes and cardiovascular alterations. The carnitine derivate, propionyl-L-carnitine (PLC), plays a key role in energy control. Our aim was to evaluate metabolic and cardiovascular effects of PLC in diet-induced obese mice.

**Methods:**

C57BL/6 mice were fed a high-fat diet for 9 weeks and then divided into two groups, receiving either free- (vehicle-HF) or PLC-supplemented water (200 mg/kg/day) during 4 additional weeks. Standard diet-fed animals were used as lean controls (vehicle-ST). Body weight and food intake were monitored. Glucose and insulin tolerance tests were assessed, as well as the HOMA_IR_, the serum lipid profile, the hepatic and muscular mitochondrial activity and the tissue nitric oxide (NO) liberation. Systolic blood pressure, cardiac and endothelial functions were also evaluated.

**Results:**

Vehicle-HF displayed a greater increase of body weight compared to vehicle-ST that was completely reversed by PLC treatment without affecting food intake. PLC improved the insulin-resistant state and reversed the increased total cholesterol but not the increase in free fatty acid, triglyceride and HDL/LDL ratio induced by high-fat diet. Vehicle-HF exhibited a reduced cardiac output/body weight ratio, endothelial dysfunction and tissue decrease of NO production, all of them being improved by PLC treatment. Finally, the decrease of hepatic mitochondrial activity by high-fat diet was reversed by PLC.

**Conclusions:**

Oral administration of PLC improves the insulin-resistant state developed by obese animals and decreases the cardiovascular risk associated to this metabolic alteration probably via correction of mitochondrial function.

## Introduction

Obesity is a fast growing problem that is reaching epidemic proportions worldwide, and is associated with an increased risk of premature death [1]. Individuals with a central deposition of adipose tissue display elevated cardiovascular morbidity and mortality, including stroke, congestive heart failure, myocardial infarction and cardiovascular death, and this is independent of the association between obesity and other cardiovascular risk factors [2], [3]. Obesity, in particular abdominal obesity, was pointed out as a primary contributor to acquired insulin resistance, as increasing adiposity is correlated with impaired insulin action, and participated to an increased occurrence of type 2 diabetes.

Propionyl-L-carnitine (PLC) is a short chain fatty acid esterified to carnitine that is rapidly transported into cells, in particular to the mitochondria, where it is transformed into free carnitine and propionyl coenzyme A [4], [5]. The latter is converted into succinyl coenzyme A and finally to succinate, which is involved in the citric acid cycle. Through this mechanism, PLC supplies energy to ischaemic tissues [4], [6]. L-carnitine plays an important role in the transport of long-chain fatty acids into the mitochondrial matrix, since it serves as an essential substrate for the enzyme which catalyses the initial step of β-oxidation, carnitine palmitoyltransferase 1. Thus, both carnitine and PLC play significant roles in fatty acid oxidation and energy expenditure. Additionally, both compounds exert protective effects on vascular endothelium [7]–[9].

Acute L-carnitine administration has demonstrated some beneficial effects on glucose disposal of patients with diabetes (for review see: [10]). However, long-term administration, whole-body effects, as well as the mechanisms leading to such effects, has been poorly evaluated. We have recently evaluated the preventive effect of PLC on obese Zucker rats as an animal model of obesity and insulin resistance [11]. In this previous study, PLC decreases body weight gain, food intake, adiposity, insulin serum concentration and triglyceride liver content and improved insulin resistance [11]. However, preventive but not PLC treatment effect, once obesity has been established, has been evaluated in our former study. Besides, the excess of plasma non esterified fatty acid (NEFA) associated with obesity has been extensively pointed out as the main cause leading to the insulin-resistant state and development cardiovascular complications secondary to chronic overweight [12], [13]. Thus, an experimental model that represents the progressive development of insulin resistance in obesity is the mouse fed with high-fat (HF) diet. Therefore, the aim of the present study was to evaluate the effects of oral administration of PLC on mice in which obesity has been already established using the HF diet in order to test the hypothesis that PLC might be used as a treatment strategy.

## Materials and Methods

### Animals and protocol design

Six week old C57BL/6 mice (Janvier, Paris, France) were fed with a HF diet −42% kcal from fat, 15.2% kcal from proteins and 42.7% kcal from carbohydrates- (TD. 88137, Harlan, Barcelona, Spain) for 9 weeks. Once diet-induced obesity (DIO) was reached, animals were divided into two groups of n = 10, receiving either water (vehicle-HF) or PLC-supplemented drinking water (200 mg kg^−1^ day^−1^, PLC-HF) during 4 weeks. Ten animals receiving a standard diet and water were used as a lean group (vehicle-ST). Body weight and food intake were controlled every three days and water intake daily monitored. The concentration of PLC contained in the drinking water was weekly readjusted, taking into account both the average daily water intake and the weekly body weight. The selected dose of PLC has been previously reported to improve endothelial function in spontaneously hypertensive rats [7]–[9] and to prevent obesity development and insulin resistance in fatty Zucker rats [10]. Additionally, this dose assures the increase of L-carnitine tissue levels since a similar dose of PLC (180 mg/kg/day) administered in the drinking water during two weeks led to an increase in the cardiac total carnitine content [14]. At the end of treatment period, animals were anesthetized with isoflurane and blood was harvested by intracardiac puncture. Lungs, spleen, kidneys, pancreas, heart, liver and epididymal, abdominal, mesenteric and subcutaneous fats were isolated and weighted.

The protocol for animal handling and experimentation of this investigation was in accordance with the European Union European Community guidelines for the ethical treatment of animals (European Economic Community Directive of 1986; 86/609/EEC) and was approved by the Ethical Committee for Animal Research of Seville University (Approval ID: 20090527).

### Glucose tolerance and insulin tolerance tests

After four weeks receiving vehicle or PLC, intraperitoneal glucose tolerance test and insulin tolerance test were performed.

The glucose tolerance test was performed by injection of glucose (2 g/kg body weight) to mice previously starved for 12 h. Blood samples were collected from the tail vein just before and at 30, 60, 90, and 120 min after the administration of glucose. Plasma glucose concentration was determined using a blood glucose commercial monitoring meter (Optimun Xceed, Abbot, Rungis, France) and reactive strips (Optium reactives strips, Abbot, Rungis, France).

The insulin tolerance test was performed by injection of insulin (0.8 UI/kg body weight, Humulina Regular, Lilly, Madrid, Spain) to mice previously starved for 4 h. Then, plasma glucose concentration was determined before and at after 30, 60, 90 and 120 min after the administration of insulin.

### Biochemical profile

Serum samples were obtained from blood by centrifugation for 15 min at 950 *g* and room temperature. Fasting glucose, insulin and NEFA were measured using commercial kits (SpinReact, Gerona, Spain; Millipore, Billerica, MA and WakoChemicals, Neuss, Germany, respectively). Triglycerides, total cholesterol, low density lipoprotein (LDL) cholesterol and high density lipoprotein (HDL) cholesterol were measured with respective enzymatic kits from Roche Diagnostics. The homeostasis model assessment to quantify insulin resistance (HOMA_IR_) was calculated as previously described [15].

### Free carnitine and acyl-carnitines analysis

Free carnitine and acylcarnitines analysis was performed by derivatization to butyl esters and flow injection electrospray ionization tandem mass spectrometry (ESI-MS/MS). Carnitine and its acyl esters were detected by looking for the precursor ions of m/z = 85 using an applied Biossytems Sciex (API 3000). Quantitative analysis was achieved by comparing the signal intensity of an analyst against the corresponding internal standard. Long chain fatty acids refer to the sum of C10 to C18.

### Systolic blood pressure

Blood pressure was measured using the non invasive tail-cuff technique three times a week. Each mouse was trained to the tail-cuff technique during a week before measurements were recorded. A Physiograph Desk Model and an Electro-Sphygmomanometer (BIOSEB; Paris, France) were used. Five separate measurements were made on conscious animals for systolic blood pressure determinations.

### Echocardiography examination

*In vivo* transthoracic echocardiography was performed using a VEVO 770 ultrasound machine from Visualsonics equipped with a 15-MHz imaging transducer in mice anesthetized with isoflurane. Briefly, a two-dimensional short axis view of the left ventricle was obtained at the level of the papillary muscle in order to record M-mode tracings. Diastolic left ventricular dimension (LVDED), systolic left ventricular dimension (LVSED) and cardiac output were measured. Cardiac index was calculated by normalizing the cardiac output to the animal body weight.

### Vascular reactivity

Thoracic aorta was dissected and placed in modified Krebs-Henseleit bicarbonate solution (KHS), final composition in mmol/l: NaCl 118, KCl 4.75, NaHCO_3_ 25, MgSO_4_ 1.2, CaCl_2_ 1.8, KH_2_PO_4_ 1.18, and glucose 11. Aortic rings (1.5–2 mm in length) were mounted on a wire myograph (Danish MyoTechnology, Aarhus, Denmark) filled with KHS. Arterial segments were stretched to a resting tension of 5 millinewtons and allowed to equilibrate for 30 min. Endothelium-dependent vasodilatation in response to acetylcholine (ACh, 1 nmol/l to 10 mmol/l) was studied in aortas with endothelium pre-contracted with the thromboxaneA_2_ agonist (9,11-dideoxy-11a, 9a-epoxymethanoprostaglandinF_2α_) U46619 at 80% of their maximal response.

Concentration-response curves were assessed in the absence and in the presence of the NO synthase (NOS) inhibitor N^ω^-nitro-L-arginine-methyl ester (L-NAME; 300 mmol/l).

### Nitric oxide (NO) determination by electron paramagnetic resonance (EPR)

Aorta, liver, skeletal muscle and heart were dissected and incubated for NO production for 30 min in Krebs–Hepes buffer containing albumin, CaCl_2_ and L-Arginine. Sodium-diethyldithiocarbamate (NaDETC) and FeSO_4_·7H_2_O were separately dissolved under argon gas bubbling in 10 mL volumes of ice-cold Krebs–Hepes buffer. These were rapidly mixed to obtain a pale yellow-brown opalescent colloid Fe(DETC)_2_ solution, which was used immediately. The colloid Fe(DETC)_2_ solution was added to vessels and incubated for 45 min at 37°C. Then, tissues were immediately frozen in plastic tubes using liquid N_2_. NO measurement was performed on a table-top x-band spectrometer Miniscope (Magnettech, MS200; Berlin, Germany). Recordings were made at 77 uK, using a Dewar flask. Instrument settings were 10 mW of microwave power, 1 mT of amplitude modulation, 100 kHz of modulation frequency, 150 s of sweep time and 3 scans [16]. Signals were quantified by measuring the total amplitude, after correction of baseline as done previously [16]. Values are expressed in unit/mg weight of dried tissue.

### Mitochondrial enzyme activities in muscles and liver homogenates

Muscles and liver homogenateswere prepared as previously described and activities of citrate synthase (CS), complex I, II, III and IV were measured spectrophotometrically at 37°C via an adaptation of the method described by Malgat et al. [17], and in agreement with the Mitochondrial Diseases Group of the Association Française de Myopathie. NADH ubiquinone reductase (complex I) was determined by monitoring the oxidation of NADH at 340 nm. Succinate ubiquinone reductase (complex II) was measured by following the reduction of 2,6-dichlorophenolindophenol at 600 nm. The assay of complex II is a function of both succinate oxidation and electron transfer to ubiquinone. Ubiquinone cytochrome c reductase (complex III) was determined by monitoring the reduction of cytochrome c at 550 nm. Cytochrome c oxidase (complex IV) activity was measured by monitoring the oxidation of reduced cytochrome c at 550 nm. CS activity was measured by monitoring the change in optical density of 5,5′-dithio-bis(2-nitrobenzoic acid) at 412 nm. Enzymatic activities are expressed in nmol of product formed per min and per mg of protein in homogenates. Protein concentrations were determined by Lowry's method.

### ATP levels in liver homogenates

ATP concentration was determined in liver homogenates by a colorimetric assay following the manufacturer-recommended protocol (Abcam, Cambrigde, UK).

### Data analysis

Data represented are means ± SEM of n = 10 animals. Relaxation induced by ACh was expressed as a percentage of the contraction. Graph-Pad Prism Software Version 5.0 (San Diego, CA) was used to calculate nonlinear regression. For glucose and insulin tolerance tests, areas under curves were analyzed in each individual incremental glucose concentration curve in order to evaluate the glycaemia reached during the whole assay period. Differences between means were assessed by one-way analysis of variance followed by Bonferroni's test, except in vascular reactivity experiments where a two-way analysis of variance was used to compare concentration–response curves with agonists. Differences were considered significant when *P*<0.05. StatView Software package Version 5.0 (SAS Institute Inc, Cary, NC) was used for statistical analysis.

### Drugs and chemicals

PLC was kindly provided by Sigma-Tau (Pomezia, Italy). All the chemicals were purchased from Sigma Chemical Co (St Louis, MO, USA).

## Results

### Food intake and body and organ weights

As expected, after receiving the HF feeding during 9 weeks, animals displayed higher values of body weight than those fed with the standard food (data not shown).

**Figure 1:**
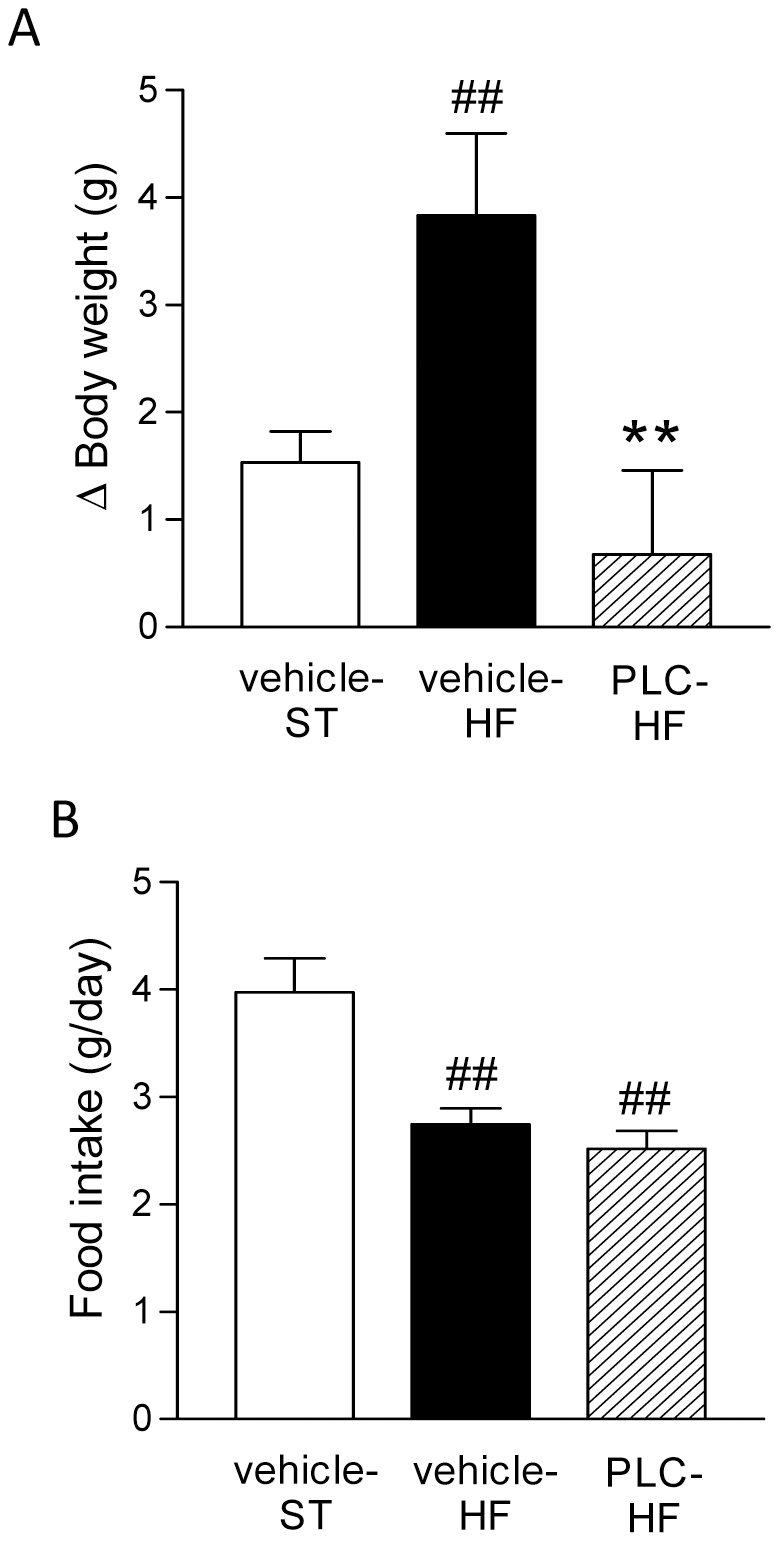
PLC treatment decreases high-fat (HF) diet-induced body weight gain without changing the food intake in mice with established diet-induced obesity (i.e. mice previously fed. A: Body weight increases during PLC treatment period (from 9^th^ to 14^th^ week). B: Daily food intake. Values are means and SEM (n = 10). ^##^ *P*<0.01, ^###^ *P*<0.001 *vs* vehicle-ST, **P<0.01 *vs* vehicle-HF.

Focusing on the PLC treatment period (i.e. next 4 weeks), it was observed a greater body weight gain in vehicle-HF *vs* vehicle-ST (Figure 1A, *P*<0.01 *vs* vehicle-ST) and PLC treatment completely blunted this increase (Figure 1A, *P*<0.01 *vs* vehicle-HF). HF groups displayed lower food intake compared to vehicle-ST group (Figure 1B, *P*<0.01 *vs* vehicle-ST). However, the effect of PLC was not due to changes in food intake between the HF groups inasmuch food intake was not significantly different between vehicle-HF and PLC-HF (Figure 1B).

The greater values of body weight found in animals fed with the HF diet was accompanied by higher weight values of liver and fat accumulated around different tissues: epididymal, abdominal, mesenteric and subcutaneous fats (*P*<0.001 *vs* vehicle-ST, Table 1). Interestingly, the increases of either liver (*P*<0.001) or visceral fat weights (epididymal-*P*<0.001-, mesenteric-*P*<0.05- and abdominal-*P*<0.01-) were lower than those of the vehicle-HF group after PLC treatment (Table 1). In contrast, no changes between weights of lean mass organs (i.e. heart, pancreas, spleen or kidneys) were found between groups (Table 1).

**Table 1:** PLC treatment decreases the weight values of liver and fat accumulated around different tissues (epididymal, abdominal, mesenteric and subcutaneous fats) induced by the HF fed animals.

	Vehicle-ST		Vehicle-HF		PLC-HF	
*Final body weight (g)*	25.6 (0.68)		41.6 (1.57)###		35.1 (1.06)### **	
	Wetweight (g)	% of body weight	Wetweight (g)	% of body weight	Wetweight (g)	% of body weight
*Heart*	0.12 (0.01)	0.57 (0.02)	0.15 (0.01)	0.58 (0.02)	0.15 (0.01)	0.59 (0.04)
*Lung*	0.18 (0.01)	0.65 (0.03)	0.19 (0.01)	0.75 (0.06)	0.17 (0.01)	0.66 (0.03)
*Spleen*	0.16 (0.09)	0.64 (0.36)	0.12 (0.01)	0.29 (0.03)	0.21 (0.08)	0.37 (0.09)
*Kidney*	0.35 (0.01)	1.36 (0.02)	0.48 (0.02)	1.16 (0.04)	0.42 (0.01)	1.21 (0.04)
*Pancreas*	0.26 (0.05)	0.90 (0.03)	0.22 (0.02)	0.85 (0.07)	0.24 (0.03)	0.92 (0.12)
*Liver*	0.96 (0.03)	3.75 (0.10)	2.62 (0.43)###	6.29 (1.36)###	1.61 (0.14)### ***	4.06 (0.56)^#^ ***
Adipose tissue:						
*Epididimal*	0.35 (0.05)	1.39 (0.21)	2.06 (0.14)###	4.95 (0.33)###	1.52 (0.09)### **	4.34 (0.29)###
*Abdominal*	0.17 (0.02)	0.66 (0.11)	0.95 (0.15)###	2.30 (0.32)###	0.74 (0.08)### *	2.13 (0.22)###
*Mesenteric*	0.17 (0.03)	0.66 (0.11)	0.73 (0.08)###	1.76 (0.19)###	0.40 (0.06)^#^ **	1.16 (0.10)*
*Subcutaneous*	0.26 (0.05)	1.02 (0.20)	2.69 (0.26)###	5.98 (0.63)###	1.93 (0.19)### **	5.50 (0.41)###

Lean organ weights are not different between the three groups. Values are means and SEM (n = 10).

### *P*<0.001 *vs* vehicle-ST,

* *P*<0.05,

** *P*<0.01 and

*** *P*<0.001 *vs* vehicle-HF.

### Plasma free L-carnitine and acylcarnitine levels

To ensure that the effects observed during oral treatment of PLC were effectively due to PLC itself but not to its metabolites, we have measured plasmatic PLC and free L-carnitine. Interestingly, animals receiving PLC treatment during four weeks showed the highest plasmatic concentrations of both free-L-carnitine and PLC (*P*<0.01 *v*s vehicle-ST and vehicle-HF and *P*<0.05 *vs* vehicle-ST and vehicle-HF, respectively, Table 2). These results provide evidence of intestinal absorption of PLC, although a partial hydrolysis of this compound cannot be excluded. Moreover, the observed effect of treatment can be accounted for both PLC and L-carnitine.

**Table 2:** PLC exerts beneficial effects on different biochemical plasma parameters evaluating the presence of insulin resistance, the lipid profile and the free- and acyl-carnitines plasma levels.

	Vehicle-ST	Vehicle-HF	PLC-HF
*Fasting glucose (mg/dL)*	110.40 (20.67)	218.10 (17.60)##	168.50 (21.34)
*Insulin (ng/mL)*	0.72 (0.13)	2.37 (0.37)###	1.15 (0.17)### ***
*HOMA_IR_*	5.28 (1.52)	31.28 (5.44)###	14.83 (2.76)### ***
*Triglycerides (mg/dl)*	102.88 (11.85)	131.65 (24.03)###	137.85 (14.10)###
*NEFA (mg/dL)*	29.53 (2.43)	41.40 (1.95)###	38.38 (1.18)#
*Cholesterol (mg/dl)*	88.39 (5.50)	202.74 (16.03)###	175.30 (11.71)### **
*HDL/LDL*	13.92 (2.52)	4.36 (0.52)##	4.60 (0.31)##
*Free LC (µmol/l)*	24.17 (2.85)	23.00(1.55)	35.75 (1.00)## **
*PLC (µmol/l)*	0.50 (0.10)	0.41 (0.03)	1.05 (0.30)# *
*LC-AC (µmol/l)*	1.45 (0.20)	0.92 (0.05)#	1.13 (0.10)

LC: L-carnitine; PLC: propionyl-l-carnitine; LC-AC: long-chain acylcarnitines. Values are means and SEM (n = 10).

# *P*<0.05,

## *P*<0.01,

### *P*<0.001 *vs* vehicle-ST,

* *P*<0.05,

** *P*<0.01 and

*** *P*<0.001 *vs* vehicle-HF.

Interestingly, untreated fatty animals displayed a lower concentration of plasmatic long-chain acylcarnitines compared to the vehicle-ST group (*P*<0.05 *vs* vehicle-ST) but not to PLC-HF group.

### Glucose tolerance and sensitivity to insulin

As shown in Table 2, HF diet induced a significant increase in fasting glucose concentrations, insulin and subsequently HOMA_IR_ compared to vehicle-ST, demonstrating the presence of the insulin resistance state. This insulin-resistance state was improved by PLC treatment since the animals receiving this carnitine derivate significantly reduced plasmatic insulin and HOMA_IR_ (*P*<0.001 *vs* vehicle-HF, Table 2) and fasting glucose was not significantly different to vehicle-ST.

**Figure 2:**
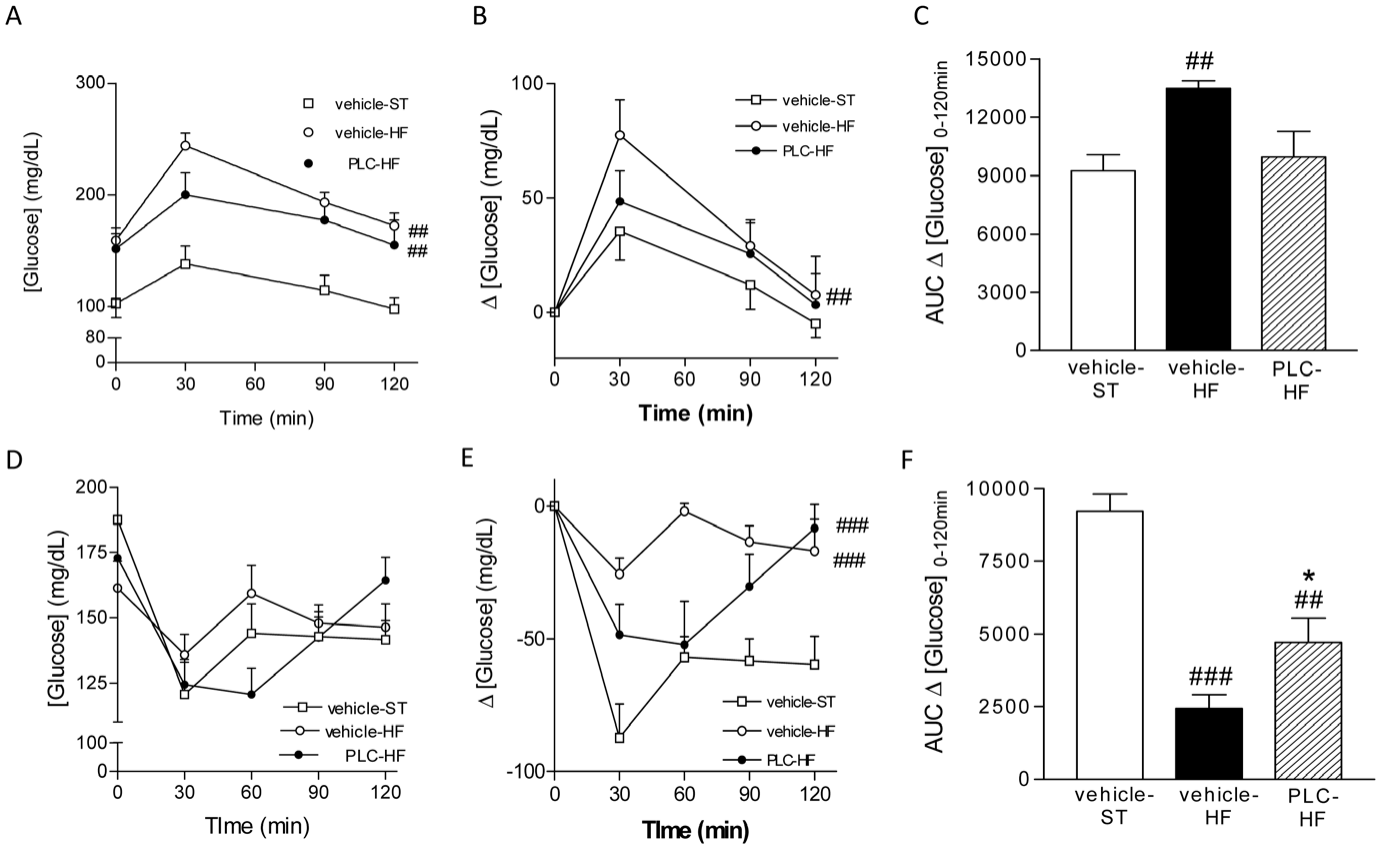
PLC restores the impaired responses to glucose overload and insulin administration induced by high-fat (HF) fed animals. A, B and C: Intraperitoneal glucose tolerance test performed in overnight fasted animals. A: Plasma glucose concentrations at baseline and at 30, 60, 90 and 120 minutes after glucose overload (2 mg/kg). B: Incremental glucose concentrations at 30, 60, 90 and 120 minutes after glucose overload. C: Area under curves describing the increase of glycaemia from 0 to 120 minutes after the glucose overload. D, E and F: Insulin sensitivity test. D. Plasma glucose concentrations at baseline and at 30, 60, 90 and 120 after insulin administration (0.8 U/kg body weight). E: Decrease of glycaemia suffered at 30, 60, 90 and 120 after insulin injection. F Area under curves describing the decrease of glycaemia from 0 to 120 minutes after the insulin administration. Values are means and SEM (n = 10). ^##^ *P*<0.01, ^###^ *P*<0.001 *vs* vehicle-ST, *P<0.05 *vs* vehicle-HF.

Such results were supported by those obtained in two functional tests, the glucose and the insulin tolerance tests. The glucose tolerance test revealed that, after receiving the oral glucose overload, animals fed with the HF diet developed post-load hyperglycaemia compared to the vehicle-ST group throughout the assay (*P*<0.001 *vs* vehicle-ST, Figure 2A). However, this glucose overload led to a significant increase of glycaemia only in the vehicle-HF (*P*<0.01 *vs* vehicle-ST, Figure 2B and C), whereas the incremental glycaemia maintained during the test remained similar in the vehicle-ST and the PLC-HF groups (Figure 2B and C).

The intraperitoneal administration of insulin led to a decrease of systemic glucose concentration in every experimental group during the insulin tolerance test (Figure 2D). As expected, both HF groups developed a blunted response of glucose concentrations following insulin injection (*P*<0.001 *vs* vehicle-ST, Figure 2D and E). Most importantly, such insulin-induced glycaemia decrease resulted significantly less damaged in the PLC-HF group compared to vehicle-HF (*P*<0.01 *vs* vehicle-HF, Figure 2F), supporting the existence of an enhanced response to insulin after PLC treatment.

### Plasmatic lipid profile

A negative lipid profile was found in obese animals treated or not with PLC compared to lean mice since they presented enhanced plasmatic concentrations of NEFA (*P*<0.01 *vs* vehicle-ST, Table 2), triglycerides, total cholesterol (*P*<0.001 *vs* vehicle-ST, Table 2) and a lower HDL/LDL ratio (*P*<0.01 *vs* vehicle-ST, Table 2). In HF-fed mice, PLC treatment did not modify the lipid profile except the reduction of total cholesterol (*P*<0.01 *vs* vehicle-HF) (Table 2).

### Mitochondrial activity in muscle or liver homogenates

HF diet resulted in alterations in liver mitochondrial function (Table 3). Activity of respiratory chain complexes I, II, III and IV was significantly lower than in the lean control group (Table 3, *P*<0.05). There was no significant difference in CS activity between vehicle-HF and vehicle-ST expressed by mg of protein in the homogenate (Table 3) or by per g wet liver. In addition expression of CS in liver by western Blot analysis with actin as reference was also similar in the two groups (data not shown) suggesting that the HF diet-dependent decrease in activity of complexes was not due to variation in mitochondrial mass. The decrease activity of mitochondrial complexes was largely prevented by treatment with PLC. Moreover, PLC treatment led to an increase in CS activity (Table 3, *P*<0.05 *vs* vehicle-HF) without modification of the expression protein level (data not shown). In contrast, we did not observe in the “red” muscle soleus any differences between the three groups. However, in the white part of the gastrocnemius muscle, activity of complex II was decreased after HF diet feeding (*P*<0.05 *vs* vehicle-ST) that was completed abolished upon PLC treatment.

**Table 3:** PLC treatment improves activity of mitochondrial respiratory chain complexes.

Complex	Vehicle-ST	Vehicle-HF	PLC-HF	P value
*Liver*				
CS	355.25±27.71	365.25±10.83	480.52±41.11*	<0.05
I	147.25±23.43	107.2±18.32#	126.20±23.91	<0.05
II	228.72±45.70	174.21±7.13#	222.21±41.02	<0.05
III	70.70±7.71	38.03±2.41#	50.11±17.51	<0.05
IV	239.23±38.22	190.72±18.51#	233.00±55.11	<0.05
*Gastrocnemius muscle*				
CS	440.82±83.61	380.11±65.95	485.06±51.80	ns
I	90.71±23.82	96.33±48.65	93.95±60.71	ns
II	59.13±11.01	44.71±11.91#	71.22±16.23	<0.05
III	269.24±37.60	290.85±33.51	352.12±76.55	ns
IV	354.53±194.40	281.51±179.13	487.15±307.10	ns
*Soleus muscle*				
CS	925.55±100.41	935.90±54.51	951.13±70.50	ns
I	487.41±95.82	470.01±130.84	393.95±58.60	ns
II	213.74±55.72	184.11±27.40	170.91±42.10	ns
III	225.50±96.41	144.25±83.65	169.54±57.21	ns
IV	620.20±213.23	654.91±340.05	647.11±60.45	ns

CS: citrate synthase. Enzymatic activities are expressed in nmol of product formed per min and per mg of protein. Values are means and SEM (n = 10).

# *P*<0.05 *vs* vehicle-ST,

* *P*<0.05 *vs* vehicle-HF.

### ATP levels in liver homogenates

To better illustrate PLC effects on hepatic energy metabolism in HF-fed animals, ATP concentration was measured in liver homogenates. According to that observed in mitochondrial activity, HF-diet induced a decrease in ATP hepatic concentration (*P*<0.05 *vs.* vehicle-ST). The ATP concentrations were 0.96±0.17 and 0.51±0.09 in control and HF-diet animals, respectively. Interestingly, PLC treatment was accompanied by the restoration of ATP levels in the liver of obese animals (0.98±0.18, *P*<0.05 *vs.* vehicle-HF).

### Tissue NO production

**Figure 3:**
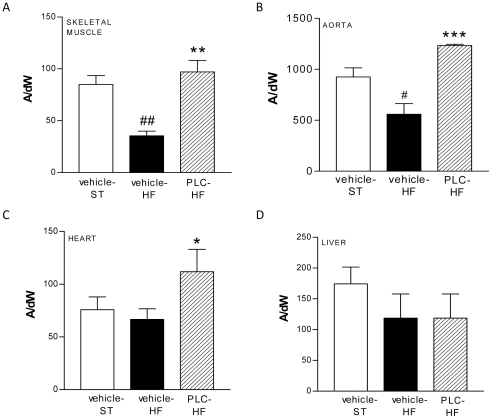
PLC increased the nitric oxide (NO) release in heart, skeletal muscle and aorta. Measurement of *in situ* NO production by electron paramagnetic resonance in skeletal muscle (A), aorta (B), heart (C) and liver (D). Values are means and SEM (n = 10). ^##^ *P*<0.01, ^#^ *P*<0.05 *vs* vehicle-ST, ***P<0.001, *P<0.05 *vs* vehicle-HF.

EPR measurement of NO showed that HF diet induced a significant decrease of NO production in the skeletal muscle and the aorta (Figure 3A and B, *P*<0.01 and *P*<0.05 *vs* vehicle-ST, respectively) but not in the heart (Figure 3C). Interestingly, PLC treatment greatly enhanced its production in these three tissues from mice receiving HF diet (Figure 3A, B and C, *P*<0.001 *vs* vehicle-HF). In the hepatic tissue a non significant decrease in NO was found in PLC-treated or not-treated HF animals in relation to the vehicle-ST group (Figure 3D).

### Cardiac function and systolic blood pressure

**Figure 4:**
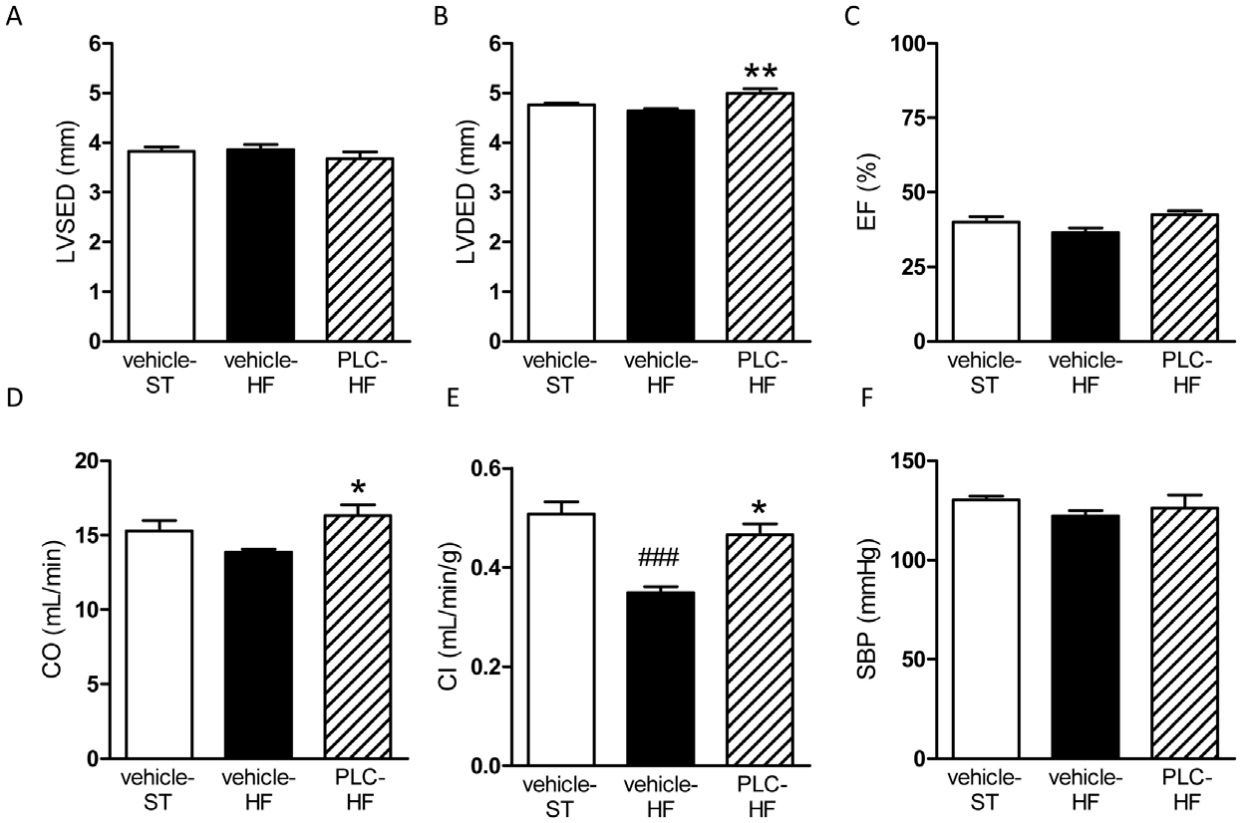
PLC improves cardiac function. A: Left ventricular systolic end diameter. B: Left ventricular diastolic end diameter. C: Ejection fraction. D: Cardiac output. E: Cardiac index (cardiac output normalized to the animal body weight). F: Systolic blood pressure. Values are means and SEM (n = 10). ^###^ *P*<0.001 *vs* vehicle-ST, *P<0.05, **P<0.01 *vs* vehicle-HF.

The effect of PLC on the structure and function of the left ventricle was assessed by echocardiography (Figure 4). We found that HF diet did not alter both LVESD and LVEDD (Figure 4A and 4B). PLC treatment slightly increased LVEDD but not LVESD in HF animals compared to vehicle-HF (*P*<0.01). Of note is that ejection fraction values were not different in the three groups (Figure 4C). Although HF diet did not significantly decrease cardiac output compared to vehicle-ST (Figure 4D), these mice exhibited decreased cardiac index when the values were normalized to the body weight (Figure 4E, *P*<0.001 *vs* vehicle-ST). Interestingly, PLC treatment significantly increased both cardiac output and cardiac index in animals receiving HF diet (Figure 4E, *P*<0.05). Finally, the systolic blood pressure did not change with diets or PLC treatment (Figure 4F).

### Aortic endothelial function

**Figure 5:**
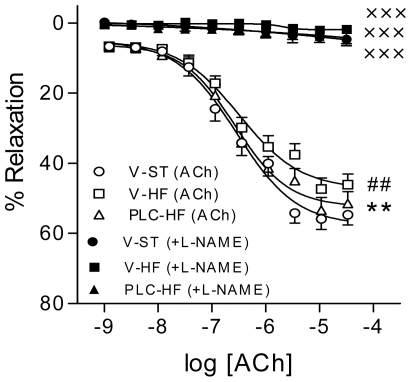
PLC improves high-fat (HF) diet-induced endothelial dysfunction. Concentration-response curves to acetylcholine (ACh, 1 nmol/l to 10 mmol/l) in the presence and in the absence of the nitric oxide synthase inhibitor N^ω^-nitro-L-arginine-methyl ester (L-NAME 300 mmol/l). Values are means and SEM (n = 10). ^XXX^ *P*<0.001 vs ACh in the absence of L-NAME, ^##^ *P*<0.01 *vs* vehicle-ST, **P<0.01, ***P<0.001 *vs* vehicle-HF.

Endothelial dysfunction, which is a key early factor in the development of atherosclerosis and a predictor of cardiovascular events, has been found in patients with obesity and metabolic syndrome. Thus, we evaluated the endothelial function in aorta from vehicle and HF animals and evaluated the potential protective effect of PLC. As shown in Figure 5, aortic rings from mice receiving HF diet displayed reduced endothelium-dependent relaxation to ACh compared to aortic rings from vehicle-ST (*P*<0.01). PLC significantly corrected the impairment in endothelium-dependent relaxation in aortas from mice receiving HF diet (*P*<0.01 *vs* vehicle-HF). In the presence of the non-selective NO synthase inhibitor, L-NAME, the endothelium-dependent relaxation to ACh was completely abolished in aortic rings from the 3 groups of animals.

## Discussion

The present study shows that PLC was able to lower the body weight gain in an experimental model of DIO, once the obesity is established. PLC improved the insulin-resistant state induced by HF feeding, likely through the restoration of a normal hepatic metabolism and lowering of tissue accumulation of toxic incompletely metabolized fatty acids. Accordingly, the improvement of the insulin sensitivity was accompanied by an enhanced NO production in skeletal muscle, heart and aorta. Additionally, PLC lowered the cardiovascular risk observed in HF-fed animals inasmuch it restored cardiac and endothelial functions, decreased visceral obesity and lowered the circulating total cholesterol.

The most important finding of the present study was the improvement of the obesity-related insulin resistance state by the PLC treatment, as shown by correction of insulinemia, HOMA_IR_ and insulin tolerance test data. We have previously found similar systemic effects in obese Zucker rats [11] and DIO mice (unpublished data) when PLC treatment was started prior to HF-diet. Here, we extent the beneficial action of PLC treatment on the insulin-resistant state even when obesity had already been well established.

PLC-treated animals displayed a decrease accumulation of ectopic visceral fat mass. This reduction of body weight and visceral adiposity was not related to a reduced food intake suggesting the existence of other mechanisms probably targeting the adipose tissue. In this sense, it has been described that the treatment of murine preadipocyte 3T3-L1 with L-carnitine reduced the content of triglycerides and total lipids after differentiation [18]. Furthermore, in mature adipocytes, L-carnitine induced the expression of genes involved in β-oxidation and lipolysis and suppression of genes involved in adipogenesis [19]. Finally, the direct effect of L-carnitine on the β-oxidation occurred in adipose tissue was recently confirmed in human preadipocytes [20]. This L-carnitine lipolytic action on adipose tissue could explain that, decreasing visceral fat accumulation, PLC treatment produced no changes in plasma levels of free fatty acids.

Ectopic visceral adiposity is the main source of inflammatory cytokines responsible for the development of obesity-induced insulin resistance [21]. Then, regardless of the mechanisms responsible for the effects of PLC treatment on visceral fat mass, its reduction may lead to the improvement of the insulin-resistant state in DIO animals. Such results are in accordance to those reported by Amin et al. [22] in which L-carnitine given in HF-induced obese rats induced a decreased of HOMA_IR_ accompanied by the loss of body weight. However, L-carnitine itself has also demonstrated some beneficial effects in insulin resistance without necessarily decreasing body weight [23], leading to the opening of extra hypothesis explaining the effects of L-carnitine and PLC on insulin resistance.

The sequence of events leading to the development of insulin resistance after starting a HF diet is still incompletely understood. Because of the central role of the liver in the whole-body energy homeostasis, liver insulin sensitivity and its potential relationship with mitochondrial oxidative phosphorylation appear to be crucial in the development of insulin resistance. Growing evidences suggest that liver rapidly respond to HF diet by metabolic and inflammatory responses that may be involved in the onset of insulin resistance [24], [25]. Alterations in the oxidative phosphorylation pathways in liver mitochondria have been reported in various rodent model of insulin resistance induced by HF diet with some conflicting results concerning respiratory chain complex activities [26], [27]. Here, we report decreased activities affecting all the complexes. Moreover, the reduced activities of complexes were not accompanied by a decrease of CS activity, supporting an intrinsic mitochondrial dysfunction rather than a lower mitochondrial number. This change in respiratory chain function was associated with a significant reduction of ATP concentration in liver homogenates indicating either a much lower ATP synthesis or a loss of the mitochondrial capacity to meet an increased energetic demand under conditions of metabolic stress such as high fat diet. Whatever the exact explanation, it is interesting to note that oral treatment with PLC led to a normalization of hepatic ATP levels and that is associated with complete correction of respiratory chain enzyme activities and with an increase in CS activity. This high citrate synthase activity might be secondary to an enhancement of specific activity since we didn't observe change in the level of protein expression. The mechanism for this is still unknown but recently a covalent modification of CS by phosphorylation/dephosphorylation was hypothesized to explain change in CS specific activity in response to insulin in cultured skeletal muscle cells [28]. Remarkably, the increased CS activity and the correction of liver mitochondrial function are associated with an improvement in peripheral insulin sensitivity as assessed by the insulin tolerance test.

Moreover, also supporting the hypothesis of an improved insulin signaling pathway secondary to PLC administration, we may well take into account the results related to NO production. Several studies have reported that insulin, in addition to its metabolic modulation, directly activates vascular endothelial and myocardial protein kinase B–endothelial NO synthase (Akt–eNOS) signaling, leading to enhance endogenous NO production. Accordingly, the deterioration of this insulin-induced NO release is frequently found in the insulin resistance state [29] and of particular interest is the increase of muscle NO availability reported in this study with PLC treatment.

The improvement of insulin sensitivity observed in treated animal with PLC could be interpreted as an indirect consequence of ameliorated liver function. Recent study demonstrated that liver by the way of the production of secretory proteins termed hepatokines may modulate the sensitivity of peripheral tissues to insulin [30]. It is also possible that PLC treatment directly exerts its beneficial effects on muscle since whole body insulin resistance depends to a great extent on muscle insulin resistance. Recently, Koves et al [31] have provided evidence that insulin sensitivity is compromised when the β-oxidation of fatty acids exceeds the capacity of the tricarboxylic acid cycle, leading to incomplete fatty acid oxidation. Here, the lower level of circulating acylcarnitines in animal fed with a HF diet may be a marker of the impaired capacity of mitochondria to export acylcarnitines resulting of the incomplete NEFA oxidation [32], [33]. In contrast, PLC treated animals showed an improved capacity for carrying out the cell these potentially toxic lipid intermediates and it is tempting to attribute the result to both the anaplerotic and the carnitine effects of the molecule. Further studies will be required to evaluate these two mutually non exclusive hypotheses.

The deterioration of the insulin-induced NO release is included between the major causes of endothelial dysfunction and cardiovascular disease related to obesity and insulin resistance [34]. Accordingly to this observation and to other previous reports, in the present work HF-feeding led to the development of endothelial dysfunction [35], [36]. As expected considering the enhanced NO release observed in the arteries from PLC-treated animals, PLC ameliorated such deteriorated arterial function.

Obesity is also a known risk factor for developing heart failure and it has been mainly related to the development of left ventricular hypertrophy and diastolic dysfunction [37]. In the present study, DIO was not related to cardiac hypertrophy. Moreover, the DIO animals did not develop hypertension, condition that is frequently necessary for the development of the obesity-induced cardiac hypertrophy. In contrast, an altered cardiac function related to obesity and insulin resistance could be pointed out as a decreased cardiac index was found in fatty animals. It is hardly to find previous reports describing such effect in the DIO models since different parameters to this cardiac index were used to evaluate the cardiac functionality (i.e. shortening fraction, LVEDD or cardiac output) [38]–[40]. However, the cardiac output is linearly related to the body weight, thus calculating the cardiac index allows a further accurate comparison between individuals with differing body weights. In fact, it has even been described the presence of enhanced cardiac output in obese individuals, whereas the cardiac index was lower in obese than in non-obese patients [41]. Interestingly, oral treatment with PLC led to an improvement of both the cardiac output and the cardiac index of DIO animals. The beneficial effects of PLC in the cardiac functionality have been deeply covered by the literature of the last decades, especially when they were evaluated in diabetic hearts [42]. Although the reduced toxic effects of incomplete β-oxidation byproducts or the enhanced NO production observed in PLC-treated animals may well be pointed out as responsible for such effects in myocardial function, the cardioprotective action of PLC in ischemic heart has also been traditionally related to an improvement of ATP production by enhancing the glucose oxidation [43]. It has been suggested that the mitochondrial export of acetylcarnitine lowers concentration of acetylCoA, which in turn favours increased pyruvate dehydrogenase activity and higher rates of glucose oxidation [43]. As a result, future studies should be driven to test the relation between pyruvate dehydrogenase activity and the beneficial effects of PLC in insulin resistance.

In conclusion, the present study provides evidence that PLC corrected the metabolic and cardiovascular alterations induced by HF feeding. Improved glucose tolerance and insulin sensitivity may participate to these beneficial effects of PLC probably via correction of the hepatic mitochondrial function and diminution of the excessive accumulation of lipid metabolism intermediates. Thus, PLC may be of potential use to treat metabolic and cardiovascular alterations associated with obesity.
